# A Comparative Study of the Lipophilicity of Metformin and Phenformin

**DOI:** 10.3390/molecules26216613

**Published:** 2021-10-31

**Authors:** Małgorzata Dołowy, Josef Jampilek, Katarzyna Bober-Majnusz

**Affiliations:** 1 Department of Analytical Chemistry, Faculty of Pharmaceutical Sciences in Sosnowiec, Medical University of Silesia in Katowice, Jagiellonska 4, 41-200 Sosnowiec, Poland; bober@sum.edu.pl; 2 Department of Analytical Chemistry, Faculty of Natural Sciences, Comenius University, Ilkovicova 6, 842 15 Bratislava, Slovakia

**Keywords:** metformin, phenformin, lipophilicity, topological indices, logP

## Abstract

The results presented in this paper confirm the beneficial role of an easy-to-use and low-cost thin-layer chromatography (TLC) technique for describing the retention behavior and the experimental lipophilicity parameter of two biguanide derivatives, metformin and phenformin, in both normal-phase (NP) and reversed-phase (RP) TLC systems. The retention parameters (R_F_, R_M_) obtained under different chromatographic conditions, i.e., various stationary and mobile phases in the NP-TLC and RP-TLC systems, were used to determine the lipophilicity parameter (R_MW_) of metformin and phenformin. This study confirms the poor lipophilicity of both metformin and phenformin. It can be stated that the optimization of chromatographic conditions, i.e., the kind of stationary phase and the composition of mobile phase, was needed to obtain the reliable value of the chromatographic lipophilicity parameter (R_MW_) in this study. The fewer differences in the R_MW_ values of both biguanide derivatives were ensured by the RP-TLC system composed of RP2, RP18, and RP18W plates and the mixture composed of methanol, propan-1-ol, and acetonitrile as an organic modifier compared to the NP-TLC analysis. The new calculation procedures for logP of drugs based on topological indices ^0^χ^ν^, ^0^χ, ^1^χ^ν^, M, and M^ν^ may be a certain alternative to other algorithms as well as the TLC procedure performed under optimized chromatographic conditions. The knowledge of different lipophilicity parameters of the studied biguanides can be useful in the future design of novel and more therapeutically effective metformin and phenformin formulations for antidiabetic and possible anticancer treatment. Moreover, the topological indices presented in this work may be further used in the QSAR study of the examined biguanides.

## 1. Introduction

Lipophilicity is one of the core factors that play a crucial role in the process of absorption, distribution, metabolism, elimination, and toxicity (ADMET properties) of bioactive compounds. Thus, the prediction of lipophilicity could be important for the determination of the pharmacokinetic profile and the pharmacological activity of new as well as already known and used drug substances [1]. Due to this fact, multiple studies are currently conducted in order to evaluate the lipophilicity of biologically active compounds and to investigate its correlation with pharmacological activity within Quantitative-Structure-Activity Relationships (QSAR) and Quantitative Structure-Property Relationships (QSPR) analysis.

Lipophilicity can be expressed by partition coefficient (P) or more often by its decimal logarithm (logP) of a compound between two phases, namely nonpolar (e.g., *n*-octanol) and polar (water). Different experimental and theoretical approaches are used for the estimation of the lipophilicity of various classes of compounds [1,2,3,4,5]. The oldest reference method used for the prediction of logP is the shake-flask method that has several limitations. This procedure is time-consuming and requires a large number of solvents and a high purity of the sample. In addition, this method allows the determination of logP in the limited range of −3.0 to +3.0. Recently, in order to overcome these difficulties, the traditional shake-flask method was successfully replaced by alternative methods such as liquid chromatography techniques, namely reversed- and normal-phase thin-layer chromatography (RP-TLC and NP-TLC) as well as reversed-phase high-performance liquid chromatography (RP-HPLC). Chromatographically determined parameters R_MW_ and logk_w_ are powerful lipophilicity descriptors [1]. Numerous studies have been performed in order to determine the lipophilic properties of different bioactive compounds as drugs or new drug candidates, including the new purine-2,6-dione derivatives, newly synthesized celecoxib analogues, thiosemicarbazides, quinolone sulfonamides, benzenesulfonohydrazides, 1,2,3-triazole-dipyridothiazine hybrids, betulin derivatives, selected steroid derivatives, chalcones, and flavonoids, by using liquid chromatographic techniques, including RP-TLC [6,7,8,9,10,11,12,13,14,15,16,17,18,19,20]. Among various chromatographic separation methods, RP-TLC is an easy-to-use and low-cost chromatographic technique. It allows analyzing several compounds simultaneously, and the consumption of organic solvents is low compared to other chromatographic techniques. In addition, the use of high-performance thin-layer chromatographic (HPTLC) plates improves the resolution and the detection sensitivity of studied compounds. The importance of TLC in the estimation of the lipophilicity of different compounds has been described in a few recently published review papers [3,4,5].

In recent years, great progress in the development of computational methods (in silico) in drug design, i.e., in the validation of drug targets and drug-like molecules, has had an impact on a significant interest in searching for new molecular descriptors such as topological indices effective in the prediction of different physicochemical properties such as lipophilicity and pharmacological activity of different groups of bioactive compounds in QSAR/QSPR studies [21,22,23,24,25,26]. A topological index is a numeric quantity associated with the chemical structure of a compound and is designed on the grounds of the molecular graph theory for the correlation with the physicochemical properties or the biological activity of compounds. The use of this descriptor allows reducing the time and the cost of the development of new drugs. Many research studies have been conducted in this area in recent years [27,28,29,30,31,32,33,34,35]. Research papers published in 2020 and 2021 confirm the promising application of selected topological indices in the design of new anticancer drugs and antiviral drugs helpful in the treatment of COVID-19 [29,30,31,32,33].

**Figure 1:**
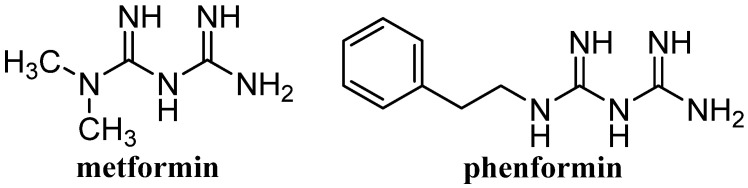
Structure of metformin and phenformin.

Our previous papers demonstrate the utility and efficiency of certain topological indices for the prediction of chromatographic retention data in various separation techniques, including the TLC method [36,37]. Some topological indices correlated well with the lipophilicity of various biologically active compounds, including pharmaceutically important compounds, such as salicylic/acetylsalicylic acid and spironolactone [36,37]. A wide application of topological indices in the prediction of the lipophilicity of active pharmaceutical ingredients is also shown in our book chapter titled Topological Indices in Modeling of Chromatographic Retention [38]. The main goal of the current paper continuing research in this field was to estimate the utility of different chromatographic conditions consisting of various stationary phases and mobile phases as well as selected topological indices for the prediction of the lipophilicity parameters of two biguanide antidiabetic compounds, metformin and phenformin (Figure 1). Both compounds contain acyclic nitrogen and should be used in stable type 2 diabetes. Metformin (dimethylbiguanide) is a well-tolerated and safe antihyperglycemic drug administrated orally as immediate-release and extended-release tablets in the treatment of type 2 diabetes mellitus [39]. Metformin’s action is based on the inhibition of hepatic gluconeogenesis. It was approved for clinical use by the FDA (Food and Drug Administration) in the United States in 1995 [39]. The latest discoveries indicate an antiaging effect and anticancer properties of metformin [40,41,42,43,44]. The long-term use of metformin in patients with diabetes is associated with lower incidence of cancer compared to patients with diabetes without metformin therapy [39]. Therefore, currently there is a growing interest in this biguanide derivative as a potential anticancer agent. As is shown in Figure 1, phenformin is a phenethyl biguanide derivative with similar activity as metformin that was used to lower blood-glucose levels but was withdrawn in some countries due to the incidence of phenformin-induced lactic acidosis [39]. Phenformin is synthesized from phenyethylamine and cyanoguanidine. It is rapidly absorbed after oral administration, and both compounds, i.e., phenformin and its metabolite, are eliminated in the urine [39].

This contribution shows for the first time the predictive power of selected topological indices for the evaluation of the lipophilic properties expressed as logP_A_ and logP_B_ of the two drugs. Due to the influence of lipophilicity on the pharmacokinetic profile of bioactive compounds including the studied biguanide drugs, the development of chromatographic systems for lipophilicity determination optimized for these compounds as well as a new calculation procedure based on topological indices can be helpful in the development of new biguanide derivatives with better bioavailability as well as the new formulations designed for novel routes of administration of these drugs. In addition to this, the topological indices calculated in this work may be helpful in a QSAR study of these biguanides.

## 2. Results and Discussion

In this work, we tested various stationary phases, i.e., chromatographic plates precoated with modified silica gel RP2, RP18, and RP18W in the reversed-phase system (RP-TLC) and plates precoated with silica gel 60F_254_ with or without a concentrating zone as well as with a mixture of silica gel 60 and Kieselguhr F_254_ and modified with a CN group (in the NP-TLC system). The mixtures consisting of an organic modifier, such as methanol, acetonitrile, propan-1-ol, and acetone, in different volume compositions, were applied as mobile phases. A wide range of organic modifier concentrations was used in this research. For most mobile phases used, they ranged from 0.40 to 0.90. The retention parameter (R_F_) obtained for two biguanides, metformin and phenformin, under different thin-layer chromatographic conditions was used to calculate the R_M_ value according to Equation (1) (see Section 3.3). Next, based on the linear dependence of R_M_ on the organic modifier content in the mobile phases used, the experimental value of lipophilicity parameters (R_MW_) of both compounds was determined according to Soczewiński–Wachtmeister’s equation, i.e., by extrapolation to 0% organic modifier concentration in the mobile phase. All obtained linear equations are presented in Table 1. It was found that the determined correlation coefficients (R) in all chromatographic systems tested were 0.99. Therefore, all equations were then successfully applied for the determination of the R_MW_ parameter as an intercept in these equations. However, the comparative analysis of the data in Table 1 shows that the lowest correlations between R_M_ and the content of the organic modifier in the mobile phase were obtained in the case of metformin studied in the reversed-phase system on RP18W (R = 0.980) and RP2 plates (R = 0.986) developed using propan-1-ol-water and plates with silica gel modified with CN groups developed using methanol-water-acetic acid (R = 0.989) in the NP-TLC system. For phenformin, all correlation coefficients were ≥0.99, except for those determined using the following chromatographic conditions: RP18 plates developed by propan-1-ol (R = 0.982) and RP2 and RP18 plates developed by acetonitrile-water in the RP-TLC system (0.905, 0.958). It can be concluded that the reason for the observed differences in the correlation coefficients in the described relationships is the interactions of stationary phases used.

The results of chromatographic lipophilicity parameter (R_MW_) values determined according to the linear equations listed in Table 1 for the investigated compounds on different stationary phases and mobile phases as well as the theoretical values of the partition coefficient as a measure of lipophilicity (logP) obtained using different calculation procedures, including the new one based on topological indices (logP_A_ and logP_B_), are presented in Table 2. The analysis of all lipophilicity parameters determined for metformin and phenformin shows higher values of this descriptor for phenformin. This fact confirms greater lipophilic properties of this compound related to metformin, which indicates in most cases much lower R_MW_ or logP values, including negative ones.

**Figure 2:**
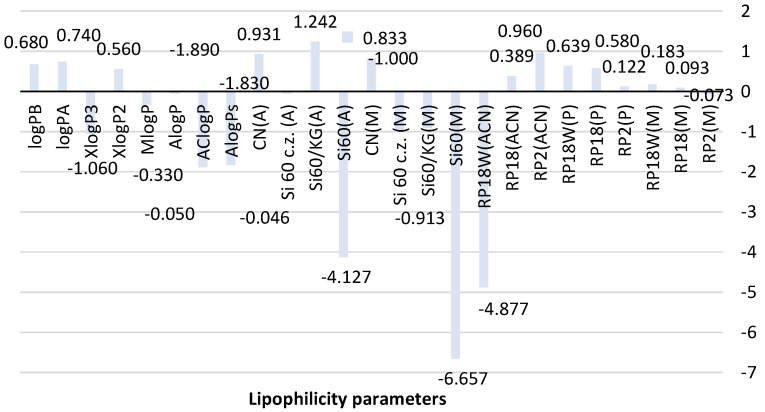
Comparison of lipophilicity parameters of metformin obtained using different methods. ACN-acetonitrile; A-acetone, M-methanol, P-propan-1-ol-organic modifier used in the mobile phase, Si60-silica gel plates, Si60c.z.-silica gel plates with the concentrating zone, Si60/KG-chromatographic plates precoated with the mixture of silica gel60 and Kieselguhr F_254_, CN-silica gel modified with cyano groups.

To estimate the utility of all chromatographic systems used in predicting the experimental value of lipophilicity denoted as R_MW_, all results obtained for metformin were compared with the theoretical values of logP predicted by using algorithms AlogPs, AClogP, AlogP, MlogP, XlogP2, and XlogP3 and also those calculated by means of a new method based on topological indices expressed as logP_A_ and logP_B_ in Figure 2.

The analysis of the theoretical lipophilicity parameters of metformin (logP) based on different calculation methods, AlogPs, AClogP, AlogP, MlogP, XlogP2, and XlogP3, in Figure 2 shows differences in the values of predicted logP depending on mathematical algorithms used to calculate them. The calculated average value of theoretical logP is −0.77 ± 0.99; thus, it shows a significant deviation. Of all presented logP values, AClogP (−1.890) and AlogPs (−1.830) indicate the similarity. However, these results have a lower value compared to logP values predicted using other calculation methods and the average logP value. The biggest similarity to the average logP value is shown by XlogP3 (−1.06). Parameters logP_A_ (0.74) and logP_B_ (0.68) newly calculated using Equations (8) and (9), see Section 3.6, are similar to XlogP2 (0.56) based on atom type and correction factor descriptors.

As shown in Figure 2, differences in the structure and physicochemical properties of stationary phases (NP-TLC and RP-TLC systems) as well as various mobile phases allowed obtaining experimental values of the lipophilicity descriptor for metformin. The R_MW_ value for metformin determined using RP-TLC systems consisting of three chromatographic plates, RP2, RP18, and RP18W, and the mixtures methanol-water (M), propan-1-ol-water (P) and acetonitrile-water (ACN) as mobile phases ranged from −4.877 to 0.960. The lowest value was obtained on RP18W plates developed with acetonitrile-water (RP18W(ACN)). The highest R_MW_ was observed on RP2 plates analyzed also using acetonitrile-water (RP2(ACN)). The reason for this difference may be associated with retention interactions exhibited by both stationary phases. The highest similarity can be seen for R_MW_ values obtained with the use of RP18 and RP2 plates and the applied mobile phases. The best correlation was observed between the chromatographic parameter of lipophilicity obtained using the mobile phase containing methanol and propan-1-ol as organic modifiers.

The analysis of R_MW_ results of metformin obtained in the NP-TLC system (Figure 2) also indicates certain differences between the lipophilicity descriptor depending on the stationary phase and the developing mixture used in this experiment. The R_MW_ obtained using the four chromatographic plates precoated with silica gel with and without a concentrating zone, coated with the mixture of silica gel and Kieselguhr, as well with silica gel modified with CN groups ranged from −6.657 to 0.833 for the mobile phase consisting of methanol-water-acetic acid and from −4.127 to 1.242 for acetone-water as a mobile phase. The biggest similarity was indicated by the data obtained on silica gel 60/Kieselguhr F_254_ plates and silica gel 60F_254_ plates with a concentrating zone developed by methanol-water-acetic acid: R_MW_ = −0.913 and −1.000.

When comparing the calculated and chromatographic parameters, it can be observed that the chromatographic plates precoated with silica gel 60 and Kieselguhr F_254_ as well as with silica gel 60 with a concentrating zone and the mobile phase consisting of methanol-water-acetic acid allowed obtaining the lipophilicity descriptor (R_MW_ = −0.913 and −1.000) that is the most similar to the average logP. This fact indicates the applicability of the presented NP-TLC system in determining the lipophilicity parameter of studied metformin. The newly calculated logP_A_ and logP_B_ are comparable to R_MW_ values obtained on RP18W (0.639) and RP18 (0.580) developed by using propan-1-ol-water in the RP-TLC system and on plates with silica gel modified with CN groups analyzed using methanol-water-acetic acid and acetone-water (0.833, 0.931).

**Figure 3:**
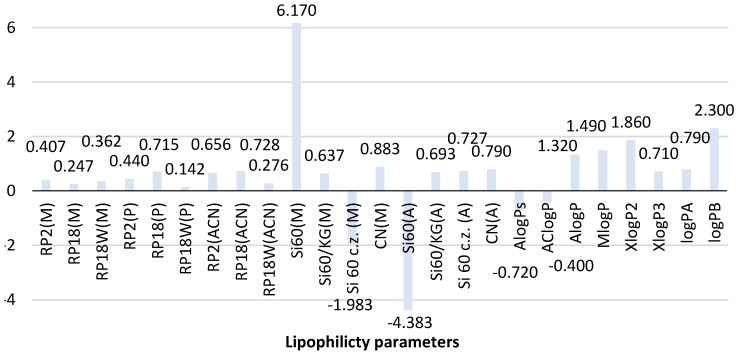
Comparison of the lipophilicity parameters of phenformin obtained using different methods. ACN-acetonitrile; A-acetone, M-methanol, P-propan-1-ol-organic modifier in the mobile phase, Si60-silica gel plates, Si60c.z.-silica gel plates with concentrating zone, Si60/KG-chromatographic plates precoated with the mixture of silica gel60 and Kieselguhr F_254_, CN-silica gel modified with cyano groups.

Figure 3 shows the comparison of theoretical and chromatographic parameters of lipophilicity determined for the second biguanide phenformin. The analysis of the theoretical value of logP obtained for this compound indicates that most predicted logP values, except for AlogPs (−0.72) and AClogP (−0.40), are positive and range from 0.71 (XlogP3) to 1.86 (XlogP2). It resulted in obtaining the average logP value equal to 0.71 ± 1.06. The greatest similarity is observed for AlogP (1.32) and MlogP (1.49). The newly calculated logP values for phenformin are 0.79 (logP_A_) and 2.30 (logP_B_). Thus, logP_A_ correlates well with the average logP. LogP_B_ calculated based on topological indices shows certain similarities to XlogP2 (1.86). This fact confirms the utility of the newly proposed calculation procedure using topological indices in predicting the theoretical value of logP of phenformin.

The further analysis of the experimentally determined chromatographic parameter of lipophilicity of this compound shown in Figure 3 indicates differences in R_MW_ values of phenformin depending on the chromatographic conditions used (stationary phase and mobile phase) in both NP and RP-TLC systems. However, the differences in the case of the reversed phase system (RP-TLC) are less compared to the NP-TLC analysis. It confirms the better suitability of RP-TLC for determining the chromatographic experimental value of the lipophilicity parameter of phenformin.

As shown in Figure 3, the chromatographic lipophilicity parameter of phenformin determined in the RP-TLC system ranged from 0.142 to 0.728. The lowest value was obtained on RP18W plates developed with the mixture of propan-1-ol-water as a mobile phase, while the highest value was determined using RP18 plates and acetonitrile-water (RP18(ACN)). The highest similarity was observed between the R_MW_ values obtained with the use of RP18 and RP2 plates and the three applied mobile phases. The smallest differences were shown by the R_MW_ values recorded for methanol-water as a mobile phase. It confirms the applicability of this mobile phase for the determination of the chromatographic lipophilicity parameter of phenformin. Taking into account the R_MW_ values determined in the NP-TLC system, it could be concluded that the influence of chromatographic conditions is much stronger compared to the described RP-TLC systems. The results were a wide range of the R_MW_ values using the four applied chromatographic plates: −1.983–6.170 for methanol-water-acetic acid and −4.383–0.790 for acetone-water as a mobile phase. The most extreme R_MW_ values compared to others were obtained using silica gel 60F_254_ and methanol-water-acetic acid (6.170) and those developed by acetone-water as a mobile phase (−4.383). Further analysis of these results indicates that the best similarity was observed for the R_MW_ values determined on silica gel modified with cyano groups (CN plates) developed in both mobile phases in NP-TLC and on silica gel 60/Kieselguhr F_254_ plates. This fact confirms the applicability of these stationary phases for the determination of the experimental value of the lipophilicity descriptor of biguanide derivatives.

The comparison of the experimental chromatographic lipophilicity parameters of phenformin with the theoretical ones, including the newly calculated logP_A_ and logP_B_, indicates that the best compatibility with the average value of the theoretical logP (0.71) and logP_A_ (0.79) is shown by the chromatographic parameters (R_MW_ values) obtained using RP18 plates and propan-1-ol (0.715) as well as RP2 (0.656) and RP18 plates developed by acetonitrile-water (0.728) in the RP-TLC system. In the case of NP-TLC analysis, logP and the logP_A_ results comparable to the average value were obtained on silica gel 60/Kieselguhr F_254_ plates and CN plates studied by methanol-water-acetic acid (0.637 and 0.883) as well as on silica gel 60F_254_ with the concentrating zone and CN plates developed by acetone-water (0.727, 0.790).

The comparison of all partition coefficients of metformin and phenformin determined by using 6 calculation methods and those obtained on the basis of newly calculated topological indices with the literature value of logP_ow_ (partition coefficient in *n*-octanol-water), which is −2.61 for metformin [45] and −0.83 for phenformin [46], indicates that of all theoretical partition coefficients, only AlogPs gave the results similar to logP_ow_ for both biguanides. Other calculation methods and the average value of all calculated logP as well as logP_A_ and logP_B_ values based on calculated topological indices for the studied compounds indicate higher differences compared to logP_ow_. This shows an important need for appropriate selection of the calculation method to predict the theoretical lipophilicity parameter for the studied biguanides. It can be stated that theoretical methods can be useful for obtaining the relative value of lipophilicity parameter only for metformin and phenformin and they cannot fully replace the classical shake-flask method. The observed differences between the certain chromatographic parameters of lipophilicity (R_MW_) and literature logP_ow_ value for both biguanides also confirm that the applied thin-layer chromatography is a rapid method allowing determining simultaneously the reliable value of the lipophilicity parameter of biguanide drugs but only under optimized chromatographic conditions.

## 3. Materials and Methods

### 3.1. Chemicals and Reagents

#### 3.1.1. Reference Standards

The pharmaceutical standards (reference samples, European Pharmacopoeia) of investigated metformin and phenformin hydrochloride were purchased from Sigma-Aldrich (St. Louis, MO, USA).

#### 3.1.2. Solvents

The mobile phases were composed of mixtures of the organic modifier-water type. The organic solvents were methanol, acetonitrile, propan-1-ol, acetone, and glacial acetic acid from POCh (Gliwice, Poland). The solvents had the HPLC grade of purity. Solutions of the two tested compounds in methanol at the concentration of 2.5 µg/µL were prepared individually.

#### 3.1.3. Chromatographic Plates

The lipophilicity study was performed on chromatographic plates for RP-TLC and NP-TLC analysis purchased from Merck (Darmstadt, Germany): silica gel RP18F_254_ (aluminum plates, No. 1.05559), silica gel RP2F_254_ (glass plates, No. 1.05747), and RP18WF_254_ (glass plates, No. 1.13142) were applied as stationary phases in the reversed-phase system. In the case of the NP-TLC system, aluminum chromatographic plates precoated with silica gel 60F_254_ without (No. 1.05554) and with a concentrating zone (No. 1.05583), glass plates precoated with silica gel 60CNF_254_ (No. 1.16464), and aluminum plates coated with the mixture of silica gel 60 and Kieselguhr F_254_ were tested (No. 1.05567).

### 3.2. Chromatographic Procedure

The lipophilicity studies of the tested compounds were carried out on chromatographic plates 10 cm × 10 cm developed using mobile phases (50 mL) prepared by mixing the respective amounts of the organic modifier (methanol/acetonitrile/propan-1-ol/acetone) and water. In the case of NP-TLC analysis, the addition of 2.4% glacial acetic acid in the mobile phase methanol-water was used. The organic modifier concentrations (volume fraction, *v*/*v*) varied in a range from 0.40 to 0.90 in constant steps of 0.10. We applied 5 μL of metformin or phenformin solution on the same chromatographic plates using precise micropipettes (5 μL, Camag, Muttenz, Switzerland). Chromatography was performed in a classical developing chamber, which was previously saturated with mobile phase vapors for 20 min. The migration distance was 7.0 cm. After the development, the plates were dried at room temperature (20 ± 1 °C) and visualized in UV light (λ = 254 nm) as the maximum wavelength for both biguanides. All analyses were repeated in triplicate. The average value of R_F_ (retardation factor) was determined in every case.

### 3.3. Chromatographic Lipophilicity Parameter (R_MW_)

In this study, the chromatographic lipophilicity parameters of both studied compounds were calculated based on the retardation factor (R_F_) obtained for metformin and phenformin in each mobile phase and chromatographic plates used as the stationary phase. The retardation factor obtained was converted into R_M_ value:

(1) $$R_{M}=\log\left(\frac{1}{R_{F}}-1\right)$$

Then, a linear correlation between the R_M_ of the tested compounds and the volume ratio of the organic solvent in the mobile phase was performed for every chromatographic plate. The extrapolation of calculated R_M_ values to the zero concentration of the organic modifier (i.e., to pure water) in accordance with Soczewiński-Wachtmeister’s Equation (2) allowed determining a valuable chromatographic parameter of lipophilicity expressed as R_MW_ [1].

R_M_ = R_MW_ − S⋅φ (2)

where R_MW_ is the chromatographic parameter obtained using Soczewiński–Wachtmeister’s Equation (2), S is the regression slope, and φ is the volume fraction of the organic modifier (e.g., methanol, propan-1-ol, acetonitrile, acetone) in the mobile phase composition. Statistica v. 13.3 software (StatSoft, Kraków, Poland) was used to determine the parameters of these linear relationships.

### 3.4. Computed Lipophilicity Parameter (logP)

The theoretical partition coefficient values—logP (AlogP_S_, AClogP, AlogP, MlogP, XlogP2, XlogP3) of the studied compounds have been obtained using the Virtual Computational Chemistry Laboratory [47], thus, through different algorithms based on atoms, molecular fragments, or molecular properties of the examined compounds described elsewhere [47]. AlogPs is the method based on the use of associative neural networks to predict the logP value from the molecular structure [48]; AClogP is an atom-additive method considering atom-type based contribution values [49]; milogP is based on group contributions, identifies a total of 220 molecular fragments, including organometallic compounds; AlogP is a classical atomic contribution approach which can be applied for neutral organic compounds [50]; MlogP is Moriguchi octanol-water partition coefficient based on quantitative structure-logP relationships using topological indexes [51]; XlogP2 is an additive atom/group model which uses about 90 basic atom types [52]; XlogP3 is a knowledge-based approach based on an additive atom/group model and the known logP value of a similar reference compound [52]. All the computed and experimental lipophilicity parameters for the tested compounds are listed in Table 2.

### 3.5. Topological Indices

Topological indices Randić (^o^χ, ^1^χ, ^o^χ^ν^, ^1^χ^ν^) and Gutman (M, M^ν^) were calculated according to the procedures sufficiently described in previous papers [53,54,55].

(3) $$M=\sum_{i=1}^{N}{{(\delta }_{i})}^{2}$$

(4) χm=∑j=1nm∏i=1m+1(δi)j−1/2

where m = 0, 1 or 2, n_m_ is the number of paths, and δ_i_ is the vertex degree in a hydrogen free graph.

Topological indices: M^ν^, ^o^γ^ν^, ^1^χ^ν^ were calculated using the valence δ^ν^ values according to the following equations:

(5) $$M^{\nu }=\sum_{i=1}^{N}{{(\delta }_{i}^{\nu })}^{2}$$

(6) χmν=∑j=1nm∏i=1m+1(δiν)j−1/2

The valence δ^ν^ or vertex degree is related to the number of valence shell electrons of the atom forming the vertex of the graph and can be expressed by the equation:

δ^ν^ = Z^ν^ − h (7)

where Z^ν^ is the number of valence electrons of the given atomic vertex, h is the number of hydrogen atoms bonded to the given vertex, ν means that the vertex degree δ has been calculated on the basis of Z. Table 3 shows the calculated topological indices for metformin and phenformin.

### 3.6. New Calculation Procedures of logP Based on Topological Indices

In this work, new procedures of logP calculation for metformin and phenformin based on newly calculated topological indices of Randić and Gutman denoted as ^o^χ^ν^,^0^χ, ^1^χ^ν^, M, and M^ν^ for both compounds were characterized by the following formulas (8) and (9):

(8) logPA=χv0χ0

(9)  logPB=χv1·MMν

## 4. Conclusions

This study confirms the relatively poor lipophilicity of the studied biguanide drugs metformin and phenformin compared to other groups of antidiabetic drugs, e.g., sulfonylureas and meglitinides. The results presented in this paper show the beneficial role of the easy-to-use and low-cost TLC technique for describing the retention behavior of the studied biguanide derivatives metformin and phenformin as known antidiabetic drugs in NP- and RP-TLC systems. To obtain a reliable value of the experimental chromatographic lipophilicity parameter (R_MW_), we needed to optimize the chromatographic conditions, i.e., stationary phase and mobile phase composition, for this study. The lowest differences in the R_MW_ values of both biguanide derivatives were ensured by the RP-TLC system composed of RP2, RP18, and RP18W plates and the mixture containing methanol, propan-1-ol, and acetonitrile as the organic modifier compared to the proposed NP-TLC analysis. Of all chromatographic conditions tested in this analysis, silica gel CN modified plates and both proposed mobile phases for NP-TLC study seem to be the most promising. This stationary phase provided smaller differences in the R_MW_ values of both drugs depending on the applied mobile phase. The comparison of theoretically determined lipophilicity descriptors denoted as logP using different software (AlogPs, AClogP, AlogP, MlogP, XlogP2, XlogP3) as well as the newly calculated logP_A_ and logP_B_ based on selected topological indices ^o^χ^ν^, ^0^χ, ^1^χ^ν^, M, and M^ν^ indicates that the different power of prediction of the applied theoretical algorithms resulted in obtaining the wide ranges of logP values for both biguanides. Therefore, the critical review of these theoretical results and comparison with the experimental values of lipophilicity descriptor (R_MW_) and logP are important to estimate reliably the lipophilic properties of the studied drugs. The new calculation procedures for logP of both drugs based on topological indices may be a good alternative to the determination by theoretical methods and thin-layer chromatography under optimized chromatographic conditions. The knowledge of the chromatographic lipophilicity parameter of both studied drugs can be useful in the future design of novel and more therapeutically effective metformin and phenformin formulations for antidiabetic and anticancer treatment. Moreover, the topological indices ^0^χ^ν^, ^0^χ, ^1^χ^ν^, M, and M^ν^ calculated in this work may be helpful in a further QSAR study of the examined biguanide drugs as potential anticancer agents.

## Figures and Tables

**Table 1:** The regression parameters (R_M_) vs. the organic modifier content in mobile phases R_M_ = R_MW_ − S⋅φ.

Chromatographic Plates	Metformin		Phenformin	
	Equation	R	Equation	R
RP-TLC system				
methanol-water				
RP2	−0.073 − 1.400⋅φ	0.999	0.407 − 2.000⋅φ	0.999
RP18	0.093 − 3.200⋅φ	0.999	0.247 − 0.689⋅φ	0.992
RP18W	0.183 − 1.300⋅φ	0.999	0.362 − 1.266⋅φ	0.995
propan-1-ol				
RP2	0.122 − 1.530⋅φ	0.986	0.440 − 2.000⋅φ	0.996
RP18	0.580 − 1.520⋅φ	0.996	0.715 − 1.770⋅φ	0.982
RP18W	0.639 − 1.930⋅φ	0.980	0.142 − 0.815⋅φ	0.997
acetonitrile-water				
RP2	0.960 − 2.700⋅φ	0.998	0.656 − 2.160⋅φ	0.905
RP18	0.389 − 1.560⋅φ	0.999	0.728 − 2.285⋅φ	0.958
RP18W	−4.877 + 0.580⋅φ	0.992	0.276 − 1.328⋅φ	0.999
NP-TLC system				
methanol-water-acetic acid				
Silica gel 60F_254_	−6.657 + 8.057⋅φ	0.999	6.170 + 7.700⋅φ	0.999
Silica gel 60/Kieselguhr F_254_	−0.913 + 0.400⋅φ	0.990	0.637 + 2.150⋅φ	0.998
Silica gel 60F_254_ with concentrating zone	−1.000 + 0.093⋅φ	0.992	−1.983 + 1.700⋅φ	0.999
CN	0.833 − 1.900⋅φ	0.989	0.883 − 1.850⋅φ	0.999
acetone-water				
Silica gel 60F_254_	−4.127 + 5.000⋅φ	0.992	−4.383 + 4.700⋅φ	0.992
Silica gel 60/Kieselguhr F_254_	1.242 − 0.707⋅φ	0.999	0.693 + 2.600⋅φ	0.999
Silica gel 60F_254_ with concentrating zone	−0.046 + 0.371⋅φ	0.998	0.727 − 2.300⋅φ	0.999
CN	0.931 − 2.343⋅φ	0.996	0.790 − 2.500⋅φ	0.990

**Table 2:** Comparison of R_MW_ values obtained with the use of different stationary phases in the RP-TLC and NP-TLC systems.

Chromatographic Plates	Metformin	Phenformin
Chromatographic Parameters (R_MW_)		
RP-TLC system		
methanol-water		
RP2	−0.073	0.407
RP18	0.093	0.247
RP18W	0.183	0.362
propan-1-ol		
RP2	0.122	0.440
RP18	0.580	0.715
RP18W	0.639	0.142
acetonitrile-water		
RP2	0.960	0.656
RP18	0.389	0.728
RP18W	−4.877	0.276
NP-TLC system		
methanol-water-acetic acid		
Silica gel 60F_254_	−6.657	6.170
Silica gel 60/Kieselguhr F_254_	−0.913	0.637
Silica gel 60F_254_ with concentrating zone	−1.000	−1.983
CN	0.833	0.883
acetone-water		
Silica gel 60F_254_	−4.127	−4.383
Silica gel 60/Kieselguhr F_254_	1.242	0.693
Silica gel 60F_254_ with concentrating zone	−0.046	0.727
CN	0.931	0.790
Theoretical values of logP		
AlogPs	−1.83	−0.72
AClogP	−1.89	−0.40
AlogP	−0.05	1.32
MlogP	−0.33	1.49
XlogP2	0.56	1.86
XlogP3	−1.06	0.71
logP_A_	0.74	0.79
logP_B_	0.68	2.30

**Table 3:** Topological indices calculated for the studied compounds.

Topological Index		Metformin	Phenformin
Gutman index	M	36	86
	M^ν^	125	174
Randić index	^o^χ	7.4393	10.5773
	^o^χ^ν^	5.4718	8.3786
	^1^χ	4.0367	6.6781
	^1^χ^ν^	2.3539	4.6565

## Data Availability

Data are contained within the article.

## Sample Availability

Samples of the compounds are available from the authors.
